# Bayesian Design for Identifying Cohort-Specific Optimal Dose Combinations Based on Multiple Endpoints: Application to a Phase I Trial in Non-Small Cell Lung Cancer

**DOI:** 10.3390/ijerph182111452

**Published:** 2021-10-30

**Authors:** Bethany Jablonski Horton, Nolan A. Wages, Ryan D. Gentzler

**Affiliations:** 1 Division of Translational Research and Applied Statistics, Department of Public Health Sciences, University of Virginia, Charlottesville, VA 22904, USA; naw7n@virginia.edu; 2 Division of Hematology/Oncology, University of Virginia Cancer Center, Charlottesville, VA 22904, USA; rg2uc@virginia.edu

**Keywords:** Bayesian trial design, early phase dose finding, treatment combinations, optimal dose combination, oncology

## Abstract

Immunotherapy and chemotherapy combinations have proven to be a safe and efficacious treatment approach in multiple settings. However, it is not clear whether approved doses of chemotherapy developed to achieve a maximum tolerated dose are the ideal dose when combining cytotoxic chemotherapy with immunotherapy to induce immune responses. This trial of a modulated dose chemotherapy and Pembrolizumab, with or without a second immunomodulatory agent, uses a Bayesian design to select the optimal treatment combination by balancing both safety and efficacy of the chemotherapy and immunotherapy agents within each of two cohorts. The simulation study provides evidence that the proposed Bayesian design successfully addresses the primary study aim to identify the optimal dose combination for each of the two independent patient cohorts. This conclusion is supported by the high percentage of simulated trials which select a treatment combination that is both safe and highly efficacious. The proposed trial was funded and was being finalized when the sponsoring company decided not to proceed due to negative findings in another patient population. The proposed trial design will continue to be relevant as multiple chemotherapy and immunotherapy combinations become the standard of care and future research will require evaluating the appropriate doses of various components of multiple drug regimens.

## 1. Introduction

The global incidence of lung cancer was 2.2 million in 2020, resulting in an estimated 1.7 million deaths [1]. In the United States, the 2021 estimated incidence of new diagnoses is 235,760 and the estimated number of deaths was 131,880. Lung cancer represents 12.4% of all new cancer cases in the US and remains the leading cause of cancer death for both men and women. Only 18% of patients are diagnosed with localized disease and an additional 22% are diagnosed with regional disease with the remaining having distant spread at the time of diagnosis. The 5-year survival for patients with localized and regional disease is 59.8% and 32.9% respectively [2].

Immune checkpoint inhibitors (ICI), such as those that inhibit programmed death ligand 1 (PD-1) or its ligand (PD-L1), have been approved as first and second-line treatments for non-small cell lung cancer (NSCLC) and are currently being evaluated in the neo-adjuvant and adjuvant setting. Pembrolizumab, a PD-1 inhibitor, is commonly used to treat patients with advanced NSCLC, either alone or in combination with chemotherapy. These agents have improved overall survival and can result in durable disease control and meaningful increases in long-term survival. Although treatment with anti-PD-1 or anti-PD-L1 antibodies can induce clinical responses in the setting of many advanced cancers, these ICIs fail to induce durable responses in a large proportion of patients. Thus, there remains a critical need to identify combinatorial approaches to augment anti-PD-1 responses and overcome immune resistance mechanisms. The efficacy of checkpoint inhibitors may be further enhanced by overcoming immune resistance mechanisms within the tumor microenvironment. Various agents have demonstrated potential to synergize with ICIs to enhance an immune response, including agents that target indoleamine 2,3-dioxygenase (IDO), vascular endothelial growth factor (VEGF), histone deacetylase (HDAC), or poly-ADP-ribose polymerase (PARP), among others. In addition, combinations of other checkpoint inhibitors (CPIs), such as those that target CTLA4, TIGIT, and LAG-3 have shown promise. In advanced NSCLC, a combination of PD-1 and CTLA4 has been shown to improve overall survival compared to chemotherapy alone and this strategy is now FDA approved [3,4].

Immunotherapy and chemotherapy combinations have proven to be a safe and efficacious treatment approach in multiple settings and there is potential to further elucidate a synergistic relationship between these modalities [5,6,7]. It is not clear whether approved doses of chemotherapy, which were developed to achieve a maximum tolerated dose (MTD), are the ideal dose when combining cytotoxic chemotherapy with immunotherapy to induce immune responses. Lower doses of chemotherapy may maximize this synergistic effect and allow for a combination with less toxicity. Further exploring the role of immunotherapy combinations with chemotherapy offers even more potential to improve response rates and survival in a disease with significant morbidity and mortality. The Checkmate 9LA study evaluated a combination of ipilimumab, a CTLA4 inhibitor, with nivolumab, a PD-1 inhibitor, with only two cycles of chemotherapy rather than the standard four cycles. This trial showed superior overall survival with this combination compared to four cycles of chemotherapy alone. However, the doses of chemotherapy given for those two cycles were at the standard dose [3].

The clinical question of interest for this study of patients with NSCLC is to explore potential benefits of lower doses of chemotherapy as well as adding a second immunotherapy agent to the commonly used standard of care regimen of platinum-doublet chemotherapy and pembrolizumab. To address this clinical question, the trial was proposed with a Bayesian design to select the optimal treatment combination by balancing both safety and efficacy of the chemotherapy and immunotherapy agents within each of two cohorts. For an overview of Bayesian design of adaptive clinical trials, we refer the reader to Giovagnoli [8] and the references therein. There are no existing dose-finding methods available to address the multitude of challenges presented by research objectives of this study. Our team adapted relevant components of existing methods to develop an appropriate and flexible design strategy. There is an increased demand to tailor early-phase clinical trial designs to the trial’s research objectives in order to treat study participants as efficiently as possible rather than reorienting the objectives to apply an “off-the-shelf” method, potentially missing the opportunity to answer promising and relevant research questions. Details of the design are provided in Section 2, followed by simulation results in Section 3. Discussion and conclusion follow in Section 4 and Section 5, respectively.

## 2. Methods

This trial is an early phase study evaluating the safety and efficacy of the combination of modulated dose chemotherapy and Pembrolizumab, with or without second immunomodulatory agent as neoadjuvant therapy for stage IB-IIIA surgically resectable NSCLC patients in two cohorts. The patient cohorts are defined by patients with adenocarcinoma (Cohort A) and squamous cell carcinoma (Cohort B). Standard histology-based chemotherapy regimens vary for the two patient cohorts, and it is not known whether one cohort is expected to have systematically greater or lesser toxicity than the other cohort. Thus, the cohorts are considered independently. Patients will receive 4 cycles of neoadjuvant combination therapy followed by surgical resection with a primary objective of determining the optimal dose combination (ODC). The ODC will incorporate both safety and efficacy and will be defined as the combination with the highest response rate among combinations with an acceptable level of toxicity. The primary outcomes guiding accrual decisions include the frequency of treatment-related dose-limiting toxicities (DLTs) and the frequency of pathologic response, assessed between 12 and 28 weeks from the start of treatment.

### 2.1. Treatment Combinations by Patient Cohort

It is anticipated that 65% of the participant population will be from Cohort A and 35% from Cohort B based on prevalence of squamous and non-squamous histology. Treatment details are provided in Table 1, where treatment combinations are labeled as Arms A1 through A6 for Cohort A and Arms B1 through B6 for Cohort B. The ODC in each cohort is the combination that is estimated to have an acceptable toxicity profile, as measured by DLTs, and a good response profile as measured by pathologic response. Adverse events are assessed and graded using the National Cancer Institute’s Common Terminology Criteria (CTCAE).

As data accumulate, each evaluable participant is classified as experiencing a DLT (yes/no) and experiencing a response (yes/no). Based on the expectedness of adverse events, the maximum allowable DLT rate is 30%. Any combination with an estimated DLT probability ≤ 30% is considered “acceptable” in terms of safety.

### 2.2. Bayesian Dose-Finding Design

The intention of this design is to determine cohort-specific ODC where treatment combination allocation is based on a Bayesian continual reassessment method accounting for both toxicity and efficacy [9]. The study is designed to accrue eligible participants using cohorts of size one. Allocation to treatment combinations is implemented for each patient cohort independently, and the process is the same in both cohorts. With regard to safety, it is assumed that increasing the dose level while holding the other agent fixed will result in an increased probability of DLT. Using this assumption, modeling incorporates a set of four possible orderings for DLT probabilities among the treatment combinations in Table 2 and a working model for DLT probabilities corresponding to the four possible orders in Table 3. This process is considered separately for each of the two patient cohorts.

The continual reassessment method (CRM) is fit for toxicity within each ordering using the working model and the accumulated data. For each working model in each cohort, m = 1, …, 4 in Table 3, the DLT probabilities are modeled using a class of one-parameter power models $\mathrm{Pr}\left(\mathrm{DLT}\;\mathrm{at}\;\mathrm{combination}\;i\right)\approx p_{mci}^{\exp(\theta_{mc})}$, where the $p_{mci}$ are the working model values for order m given in Table 3, i indexes the dose combination and c indexes the cohort.

DLT probability estimation embodies characteristics of the continual reassessment method (CRM) [10], so we use its features to specify design parameters. The skeleton values for toxicity were selected using to the algorithm of Lee and Cheung [11], using recommended specifications that yield good operating characteristics. CRM designs have been shown to be robust and efficient with the use of “reasonable” skeletons, where adjacent values have adequate spacing. The algorithm is available as a function, getprior, within the R [12] package dfcrm [13] and requires a spacing measure ρ to generate reasonable spacing between adjacent combinations in the skeleton. Simulation results in Lee and Cheung [11] indicate that the optimal range of ρ is [0.04, 0.10] for common target toxicity rates (i.e., 0.20–0.33). The value ρ=0.04 lies in the optimal range and provides a set of reasonably spaced skeleton values. The skeletons should represent the various possible orderings of regimen–toxicity curves, according to the toxicity assumptions displayed in Table 2. The class of skeletons in Table 3 was generated using the algorithm and the locations of these values were adjusted to correspond to the six orderings in Table 2 using the getwm function in R package pocrm [14].

The prior distribution on the parameter θ for all working models is given by g(θ)=N(0,0.48), a normal distribution with mean 0 and standard deviation 0.48. The standard deviation for the prior distribution was chosen according to Algorithm 9.1 in Cheung [15] using values of $\sigma_{\theta }^{LI}=0.75,\;\lambda_{1}=0.6,\lambda_{2}=1.4$ and a grid width of 0.03. According to Cheung [15], there are two practical advantages for choosing a normal distribution in this setting. First, posterior computations using Gauss–Hermite quadrature [16] under the above parametrization are accurate, and the second, Bayesian CRM utilizing a class of one-parameter models that includes the power model is invariant to the mean of a prior that forms a location-scale family. This property allows for the prior mean to be zero and the prior to be completely specified by its standard deviation, simplifying the process of calibration. A uniform prior distribution, τ(m)=1/m, is placed on each working model for each cohort so that all working models are considered equally likely a priori. Based on the observed toxicity data $D_{c}=\left\{\left(y_{ci},n_{ci}\right);\;i=1,\ldots ,6\right\}$, where $y_{ci}$ is the number of DLTs, $n_{ci}$ is the number of subjects treated on combination i, and c specifies the cohort. The likelihood for ordering m is given by

(1) $$L_{m}\left(D_{c}|\theta \right)\propto \overset{6}{\underset{i=1}{\prod }}{\left(p_{mci}^{\exp(\theta_{m})}\right)}^{y_{ci}}{\left(1-p_{mci}^{\exp(\theta_{m})}\right)}^{n_{ci}-y_{ci}}$$

Using Bayes theorem, the posterior probability for each working model given the data can then be calculated as

(2) $$P\left(m|D_{c}\right)=\frac{\tau \left(m\right)\int L_{m}\left(D_{c}|\theta \right)g\left(\theta \right)d\theta }{\sum_{m=1}^{5}\tau \left(m\right)\int L_{m}\left(D_{c}|\theta \right)g\left(\theta \right)d\theta }$$

After accrual of each participant into the trial the model associated with the largest posterior probability is selected and the DLT probability estimates, ${\hat{\pi }}_{ci}$, are updated using the chosen working model using the Bayesian form of the CRM [9] so that

(3) $${\hat{\pi }}_{ci}=\frac{\int p_{mci}^{\exp(\theta_{m})}L_{m}\left(D_{c}|\theta \right)g\left(\theta \right)d\theta }{\int L_{m}\left(D_{c}|\theta \right)g\left(\theta \right)d\theta }$$

If a tie occurs between the posterior model probabilities of two or more models, then the selected model would be randomly chosen from among the tied models. The estimated DLT probabilities are used to define a set of “acceptable” combinations with regard to safety. The maximum tolerated dose combination (MTDC) is defined as the combination with estimated DLT probability closest to the maximum allowable DLT rate of 30%. Any combination with estimated DLT rate less than or equal to that of the MTDC would be considered acceptable in terms of safety.

The probability of response $\delta_{ci}$ at combination i in cohort c  is modeled using a beta-binomial model

(4) $$z_{ci}|\delta_{ci}\sim \mathrm{Binomial}(\delta_{ci});\;\delta_{ci}\sim \mathrm{Beta}\left(\tau_{ci},\nu_{ci}\right)$$

where $\mathrm{Beta}\left(\tau_{ci},\nu_{ci}\right)$ is a beta distribution with parameters $\tau_{ci}\;$ and $\nu_{ci}$. Based on the number of responses $z_{ci}$ and the number of treated participants $n_{ci}$ on combination i in cohort c, the posterior distribution of $\delta_{ci}$ follows a beta distribution so that

(5) $$\delta_{ci}|(z_{ci},n_{ci})\sim \mathrm{Beta}(\tau_{ci}+z_{ci},\nu_{ci}+n_{ci}-z_{ci})$$

Using a non-informative Beta(0.5, 0.5) prior distribution in each cohort, the probabilities of pathologic response for each combination are estimated based on the posterior mean ${\hat{\delta }}_{ci}=(z_{ci}+0.5)/\left(n_{ci}+1\right)$, separately for each cohort. Once the set of acceptable combinations is determined in each cohort, the recommended combination varies depending on how many participants have entered the study to that point. For the first third of the trial (1/3 the maximum sample size), the combination recommendation in each cohort is based on randomization using a weighted allocation scheme. The recommended combination for the next entered participant is chosen at random from the set of acceptable combinations, with each acceptable combination weighted by its estimated response probability. Based on the estimates ${\hat{\delta }}_{ci}$, we calculate the randomization probability

(6) $$R_{ci}=\frac{{\hat{\delta }}_{ci}\;}{\sum {\hat{\delta }}_{ci}}$$

and randomize the next participant in cohort c to an acceptable combination  i with probability $R_{ci}$. This approach allows for acceptable combinations with higher estimated response probabilities to have a higher chance of being randomly chosen as the next recommended combination. For the latter two-thirds of the trial (final 2/3 of maximum sample size), the recommended combination for the next entered participant is defined as the acceptable combination with the highest estimated response probability so that the next participant is assigned the combination i satisfying $\arg\max{\hat{\delta }}_{ci}$. As each participant enters the study, a new recommended combination is obtained, and the next entered participant would be allocated to the updated recommended combination. The trial is designed to stop once sufficient information about the optimal combination in each cohort is obtained, according to the stopping rules defined in the following section.

### 2.3. Sample Size and Stopping Rules

The maximum target sample size is 60 based on obtaining sufficient information to determine the optimal dose combination in each cohort, which is defined by the combination with the highest response rate among combinations with acceptable toxicity. Stopping rules are incorporated for both safety considerations and efficient use of participants by stopping accrual to a cohort once a sufficient number of patients are treated at the ODC. If the set of acceptable combinations is empty at any point, accrual to the study will be halted. This stopping guideline will trigger a review by the study investigators and DSMC to determine if the study should be modified or permanently closed to further accrual. Accrual to the study for a cohort will end if the recommended treatment combination for the next participant is to a combination that already has 12 patients treated at that combination. If occurring, this treatment combination is determined to be the optimal dose combination for the cohort. Otherwise, accrual will continue until 60 patients are accrued to the study.

Twelve patients receiving the optimal combination will allow for adequate data to assess the pathologic response rate. Based on a Beta(0.5, 0.5) prior, if 5 out of 12 patients receiving the ODC experience pathologic response, then the posterior distribution of $\delta_{ci}$ is Beta(5.5, 7.5) according to Equation (5). The probability that the response rate for the optimal combination exceeds the standard of care is given by,

(7) $$\mathrm{Pr}\left(\delta_{ci}>0.28|z_{ci}=5,\;n_{ci}=12\right)$$

(8) $$=\int_{0.28}^{1}\frac{\Gamma \left(13\right)}{\Gamma \left(5.5\right)\Gamma \left(7.5\right)}\delta_{ci}^{4.5}{\left(1-\delta_{ci}\right)}^{6.5}$$

(9) ≈0.853,

where i and c  indicate the combination and cohort, respectively.

## 3. Simulation Results

A simulation study provides operating characteristics that convey the design’s ability to address the aims of the study. In dose-finding clinical trials, operating characteristics provide the scientific justification for the selected design and sample size, similar to that of a power analysis in a phase III clinical trial [17].

### 3.1. Design of Simulation Study

Simulations were run in R to display the performance of the design described in Section 2, with results presented in Table 4 and Table 5. Six scenarios are considered, allowing for a broad range of possible relationships between treatment dosage, DLT, and efficacy rates. In each scenario, 1000 simulated trials were run. For each treatment combination, Table 4 presents the true DLT and efficacy rates (row 1), percentage of selection as the ODC (row 2), and the average number of participants treated (row 3). In Table 4, optimal combinations are indicated in bold type, and unsafe combinations are indicated in red type. Table 5 displays the average sample size overall and by cohort. While the overall maximum sample size is 60 participants, it is assumed that 65% of participants are diagnosed with adenocarcinoma, and the remaining 35% are diagnosed with squamous cell carcinoma. This provides maximum sample sizes of 39 and 21 for Cohorts A and B, respectively. The following six scenarios were chosen to display the operating characteristics for this design, providing a wide variety of dose-toxicity-efficacy relationships.

1. All doses are safe. Intermediate chemo dose maximizes efficacy.
2. All doses are safe. More chemo yields better efficacy.
3. Highest chemo dose with immune agent 2 is unsafe. More chemo yields better efficacy.
4. Highest chemo dose with immune agent 2 is unsafe. Intermediate chemo dose maximizes efficacy.
5. Highest chemo dose with and without immune agent 2 are unsafe. Intermediate chemo dose maximizes efficacy.
6. Two cohorts have different safety and efficacy profiles.

### 3.2. Sample Size and Accrual

Accrual to the study for a cohort was designed to end once the next recommendation is to assign the next participant to a combination that already has 12 patients treated at that combination. Accrual is estimated to be 2–3 patients per month, allowing for accrual to be complete within two years. If the minimum follow-up period for participants already on study is not satisfied at the time a new participant is ready to be put on study, then the participant may be accrued to any combination by random allocation, which has accrued at least one participant and is in the acceptable safety set. At the time of combination allocation for the next participant, model-based estimates are calculated for both DLT and response probabilities using the available observed data from all participants accrued to the study at that time. It is important to note that in this design approach, some model-based decisions may be made using slightly less efficacy data than DLT data due to the longer minimum observation window for efficacy. Adjusting for 10% dropout and ineligibility, the maximum sample size should not exceed 67 patients.

### 3.3. Summary of Operating Characteristics

The selected design performs well by providing a high rate of ODC selection in the optimal combinations and a low rate of ODC selection in less desirable treatment combinations, either because of safety concerns or insufficient efficacy. Consider Scenario 1 in Cohorts A and B, where the optimal combination is the treatment combination of immune agent 2 and the intermediate dosage of chemotherapy (indicated in bold type). While all treatment combinations are considered safe, three treatment combinations with low DLT rates and high rates efficacy are highlighted in gray. For Cohort A, these three treatment combinations comprise more than 70% of the recommended ODCs while treating, on average, 58.7% of the trial participants. In Cohort B, these three treatment combinations comprise 69.9% of the recommended ODCs while treating, on average, 59.6% of the trial participants. In contrast, consider Scenario 4, where the treatment combination with immune agent 2 and the highest level of chemotherapy is unsafe. Treatment combinations with the intermediate dosage of chemotherapy, both with and without immune agent 2, have the highest level of efficacy as well as acceptable toxicity. Very few simulated trials resulted in an ODC recommendation of the unsafe treatment combination (1.7% for both Cohorts A and B). The two optimal treatment combinations comprise 76.8% and 74.7% of the ODC recommendations for Cohorts A and B, respectively. Additionally, more than half of simulated trial participants are treated on the two optimal combinations (56.1% and 60.1% for Cohorts A and B, respectively).

The maximum sample size is 60 eligible participants; however, the simulation results in Table 5 indicate that across all scenarios considered, the maximum average trial size is 46 participants. The design used for the trial both performs well and uses resources efficiently by stopping the study once the design recommends a treatment combination in which 12 participants have been treated in the cohort.

## 4. Discussion

The design for this study was chosen by balancing the primary study aims and adaptation of existing methods in developing a flexible design strategy. Careful selection of the dose-finding method allows the study design to address the primary study aim without reorienting the study goals to fit a simpler design. In this case, the primary aim of the study is to identify the ODC for each of the two independent patient cohorts. Simulation studies are provided to evaluate the operating characteristics of this design and highlight the ability of the design to identify the ODC and other desirable treatment combinations in a high percentage of trials. Additionally, simulations guide the anticipated final sample size needed to draw meaningful conclusions about the efficacy of the selected ODC.

Treatment regimens varied for the two patient cohorts, and it was not anticipated that either cohort would have systematically greater or lesser toxicity than the other cohort. Because of this, the cohorts were considered independently. If prior information indicated that one cohort was expected to have greater or lesser toxicity, appropriate changes in the design would have been made to use this order information in identifying the ODC for each cohort. Study design options for treatment combinations are limited, especially when considering the additional complexity of clinical aims. While several methods are available to account for two or more groups of participants, these designs consider dose-finding for a single agent [18,19,20,21]. The approach outlined in this paper to tailor the study design to the complex research objectives provides the framework that demonstrates how adaptive designs can be modified within a single trial to address the objectives specific to the study while advancing early development of novel treatment regimens. This manuscript aims to provide an example for designing complex early-phase trials with multiple objectives in various cohorts.

## 5. Conclusions

This phase I study design aims to identify the optimal dose combination for each of two cohorts of patients with non-small cell lung cancer, based on multiple endpoints. Simulation studies indicate that the design is well suited to address the study aims while conserving study resources. 

During the finalization of this trial protocol, the company sponsoring the study decided not to move forward with this trial due to recent negative findings in another patient group [22]. While this trial was not initiated, plans were near completion and this example highlights the benefits of using a Bayesian design for early phase clinical trials.

As multiple chemotherapy and immunotherapy combinations become the standard of care, future research will likely require evaluating the appropriate doses of the various components of the multiple drug regimen. The Bayesian phase I design described here allows for evaluation of both safe and efficacious doses for various drug combinations commonly used in NSCLC and incorporates standard histology-based chemotherapy regimens in the same trial.

## Figures and Tables

**Table 1:** Treatment combinations by cohort.

Cohort A: Adenocarcinoma Patients				
Pembrolizumab 200 mg IV Q3W				
		**Chemotherapy (mg/m^2^)**		
	Cisplatin	25	50	75
	Pemetrexed	150	375	500
Immune agent 2	Dose level 1	A4	A5	A6
	0	A1	A2	A3
**Cohort B: Squamous Cell Carcinoma Patients**				
**Pembrolizumab 200 mg IV Q3W**				
		**Chemotherapy (mg/m^2^)**		
	Cisplatin	25	50	75
	Gemcitabine	400	800	1200
Immune agent 2	Dose level 1	B4	B5	B6
	0	B1	B2	B3

**Table 2:** Possible orders of DLT probabilities.

Order (m)	Combination
1	1–2–4–3–5–6
2	1–2–4–5–3–6
3	1–4–2–5–3–6
4	1–4–2–3–5–6

**Table 3:** Working model of DLT probabilities under each ordering.

	Combination					
Order (m)	1	2	3	4	5	6
1	0.03	0.10	0.15	0.10	0.22	0.30
2	0.03	0.05	0.22	0.10	0.15	0.30
3	0.03	0.10	0.22	0.05	0.15	0.30
4	0.03	0.10	0.15	0.05	0.22	0.30

**Table 4:** Operating characteristics for Cohorts A and B.

Row 1: (True % DLT, True % Efficacy); Row 2: % Selection as OTC; Row 3: Average Participants Treated.	Immune Agent 2	Cohort A			Cohort B		
		Cisplatin, Pemetrexed (mg/m^2^)			Cisplatin, Gemcitabine (mg/m^2^)		
		25, 150	50, 375	75, 500	25, 400	50, 800	75, 1200
Scenario 1: All doses are safe. Intermediate chemo dose maximizes efficacy.	Dose 1	(0.03, 0.35)	(0.08, 0.50)	(0.20, 0.45)	(0.03, 0.35)	(0.08, 0.50)	(0.20, 0.45)
		11.4	33.4	18.7	13.0	30.3	18.1
		3.9	5.8	4.2	3.2	4.4	2.7
	0	(0.01, 0.25)	(0.05, 0.40)	(0.15, 0.35)	(0.01, 0.25)	(0.05, 0.40)	(0.15, 0.35)
		4.7	21.5	10.2	6.7	20.8	11.1
		3.1	5.3	3.7	2.6	4.3	2.6
Scenario 2: All doses are safe. More chemo yields better efficacy.	Dose 1	(0.03, 0.55)	(0.08, 0.67)	(0.20, 0.78)	(0.03, 0.55)	(0.08, 0.67)	(0.20, 0.78)
		9.5	19.9	30.9	14.1	21.0	24.6
		3.5	4.4	5.0	3.1	3.3	3.3
	0	(0.01, 0.45)	(0.05, 0.57)	(0.15, 0.68)	(0.01, 0.45)	(0.05, 0.57)	(0.15, 0.68)
		4.3	13.2	22.2	5.7	16.5	18.1
		2.9	4.7	4.5	2.3	4.2	3.1
Scenario 3: Highest chemo dose with immune agent 2 is unsafe. More chemo yields better efficacy.	Dose 1	(0.22, 0.55)	(0.27, 0.67)	(0.32, 0.78)	(0.22, 0.55)	(0.27, 0.67)	(0.32, 0.78)
		22.2	16	5.9	20	17.8	6.7
		5.2	3.9	1.5	3.8	3.2	1.3
	0	(0.20, 0.45)	(0.25, 0.57)	(0.30, 0.68)	(0.20, 0.45)	(0.25, 0.57)	(0.30, 0.68)
		12.7	28	15.2	12.2	28.3	14.7
		4.2	6.5	3.8	3.1	5.6	2.7
Scenario 4: Highest chemo dose with immune agent 2 is unsafe. Intermediate chemo dose maximizes efficacy.	Dose 1	(0.22, 0.60)	(0.27, 0.85)	(0.32, 0.70)	(0.22, 0.60)	(0.27, 0.85)	(0.32, 0.70)
		9	19.3	1.7	8.6	15.8	1.7
		3.7	4.2	1	2.5	2.7	0.7
	0	(0.20, 0.55)	(0.25, 0.83)	(0.30, 0.68)	(0.20, 0.55)	(0.25, 0.83)	(0.30, 0.68)
		6.7	57.5	5.8	10.3	58.9	4.5
		3.3	9.2	2.5	2.7	8.6	1.6
Scenario 5: Highest chemo dose with/out immune agent 2 is unsafe. Intermediate chemo dose maximizes efficacy.	Dose 1	(0.10, 0.70)	(0.22, 0.85)	(0.42, 0.70)	(0.10, 0.70)	(0.22, 0.85)	(0.42, 0.70)
		12.6	26.8	1.6	10.9	23.2	1.7
		5.2	4.2	1.2	2.7	3.5	0.8
	0	(0.08, 0.65)	(0.20, 0.83)	(0.40, 0.68)	(0.08, 0.65)	(0.20, 0.83)	(0.40, 0.68)
		7.5	47.5	4	9.7	50.7	3.8
		3.5	8.3	2.5	2.7	7.8	1.5
Scenario 6: Two cohorts have different safety and efficacy profiles.	Dose 1	(0.03, 0.55)	(0.08, 0.67)	(0.20, 0.78)	(0.10, 0.70)	(0.22, 0.85)	(0.42, 0.70)
		7.4	20.5	33.7	13.5	19.6	2
		2.6	4.6	4.6	2.8	3.1	0.8
	0	(0.01, 0.45)	(0.05, 0.57)	(0.15, 0.68)	(0.08, 0.65)	(0.20, 0.83)	(0.40, 0.68)
		3	13.6	21.8	11	49.3	4.6
		3.2	4.6	5.5	2.7	7.2	1.6

**Table 5:** Average sample size across simulations.

Scenario	Cohort A	Cohort B	Overall
1	25.4	19.8	45.2
2	24.8	19.2	44
3	25.1	19.7	44.8
4	23.9	18.8	42.7
5	24.9	19	43.9
6	25.1	18.2	43.3

## Data Availability

Not applicable.

## Abbreviations

CPI	Checkpoint inhibitor
DLT	Dose-limiting toxicity
HDAC	Histone deacetylase
ICI	Immune checkpoint inhibitor
IDO	Indoleamine 2,3-dioxygenase
MTD	Maximum tolerated dose
NSCLC	Non-small cell lung cancer
ODC	Optimal dose combination
PARP	Poly-ADP-ribose polymerase
PD-1	Programmed death-1
PD-L1	Programmed death ligand-1
VEGF	Vascular endothelial growth factor
